# Endoscopic Endonasal Supraoptic and Infraoptic Approaches for Complex “Parasuprasellar” Lesions: Surgical Anatomy, Technique Nuances, and Case Series

**DOI:** 10.3389/fonc.2022.847250

**Published:** 2022-05-26

**Authors:** YouYuan Bao, YouQing Yang, Lin Zhou, ShenHao Xie, Xiao Wu, Han Ding, Jie Wu, Limin Xiao, Le Yang, Bin Tang, Tao Hong

**Affiliations:** Department of Neurosurgery, The First Affiliated Hospital of Nanchang University, Nanchang, China

**Keywords:** endoscopic endonasal approach, parasuprasellar area, anterior clinoid process, optic canal, internal carotid artery, surgical technique

## Abstract

**Objective:**

The surgical management of lesions involving the lateral area of the suprasellar region, including the lateral aspect of the planum sphenoidale and the tight junction region of the optic canal (OC), anterior clinoid process (ACP), and internal carotid artery (ICA) and its dural rings, is extremely challenging. Here, these regions, namely, the “parasuprasellar” area, are described from the endonasal perspective. Moreover, the authors introduce two novels endoscopic endonasal supraoptic (EESO) and endoscopic endonasal infraoptic (EEIO) approaches to access the parasuprasellar area.

**Methods:**

Surgical simulation of the EESO and EEIO approaches to the parasuprasellar area was conducted in 5 silicon-injected specimens. The same techniques were applied in 12 patients with lesions involving the parasuprasellar area.

**Results:**

The EESO approach provided excellent surgical access to the lateral region of the planum sphenoidale, which corresponds to the orbital gyrus of the frontal lobe. With stepwise bone (OC, optic strut and ACP) removal, dissociation of the ophthalmic artery (OA) and optic nerve (ON), the EEIO approach enables access to the lateral region of the supraclinoidal ICA. These approaches can be used independently or in combination, but are more often employed as a complement to the endoscopic endonasal midline and transcavernous approaches. In clinical application, the EESO and EEIO approaches were successfully performed in 12 patients harboring tumors as well as multiple aneurysms involving the parasuprasellar area. Gross total and subtotal tumor resection were achieved in 9 patients and 1 patient, respectively. For two patients with multiple aneurysms, the lesions were clipped selectively according to location and size. Visual acuity improved in 7 patients, remained stable in 4, and deteriorated in only 1. No postoperative intracranial infection or ICA injury occurred in this series.

**Conclusions:**

The EESO and EEIO approaches offer original treatment options for well-selected lesions involving the parasuprasellar area. They can be combined with the endoscopic endonasal midline and transcavernous approaches to remove extensive pathologies involving the intrasellar, suprasellar, sphenoid, and cavernous sinuses and even the bifurcation of the ICA. This work for the first time pushes the boundary of the endoscopic endonasal approach lateral to the supraclinoidal ICA and ON.

## Introduction

With advances in surgical anatomy and endoscopic technology, the endoscopic endonasal approach (EEA) has been widely applied for ventral skull base lesions over the last several decades (1–3). Furthermore, this approach has been expanded to the lateral skull base, accompanied by the introduction of endoscopic transpterygoid route, such as the cavernous sinus (CS), pterygopalatine fossa and infratemporal fossa (4–9). The anatomy and related surgical nuances of these complex skull base areas have been well documented in a considerable amount of literature. Nevertheless, few reports exist on the detailed anatomy of the lateral area of the suprasellar region, including the lateral aspect of the planum sphenoidale and the tight junction region of the optic canal (OC), the anterior clinoid process (ACP), and the internal carotid artery (ICA) and its dural rings that fix its course (10, 11). There are essentially two reasons for such limited data: 1) limited access to these regions due to obstruction of vital neurovascular structures such as the optic nerve (ON) and ICA; 2) consideration of these regions as off-limits due to the lateral-seated location and intrinsic anatomical complexity. In fact, it is difficult to imagine the existence of such a high density of neurovascular and osseous as well as dural structures in such a narrow anatomical space.

Although not common, some different pathologies can afflict these regions, including primary lesions, such as ACP meningiomas and paraclinoid aneurysms, but more secondary tumors spread, such as tuberculum sellae meningiomas, invasive pituitary adenomas and craniopharyngiomas, from nearby regions. Pathologies encountered in these areas are typically intra- and extracranially. Moreover, these lesions tend to displace the ON from above and/or below, erode osseous and dural structures, and even encase the ICA and its bifurcation. Therefore, effective resection of these lesions poses a considerable challenge, even for skilled and experienced neurosurgeons.

Several traditional transcranial approaches (TCAs), including the standard or extended pterional approach (12, 13), orbital-zygomatic approach (14) and supraorbital approach (15), for accessing lesions in these areas have been described. Although TCAs can be good alternatives for subdural lesions, extradural lesions involving the intrasellar, sphenoid sinus and even CS are extremely difficult to manage because surgical corridors are inconsistent with the axis of tumor growth. In addition, inevitable brain retraction, extensive bone removal, and easy damage to important neurovascular elements make TCAs less favorable options.

In contrast, the EEA provides a direct corridor to access extradural lesions, with the advantage of easy removal of extensively involved osseous architectures and dural attachments. Additionally, a corridor to the subdural lesion is established when the lesion is removed. Most importantly, EEA allows for early identification and control of the paraclinoidal ICA, which is the main structure that must be crossed to expand laterally into these regions. These advantages are particularly promising for treating lesions involving these areas. Nevertheless, there are few reports regarding the endoscopic anatomy and how to effectively manage lesions involved these areas (10, 11).

For this reason, we sought to undertake a thorough anatomical description of the lateral aspect of the planum sphenoidale and the tight junction region of the OC, ACP, and ICA and its dural rings. These regions are located in the lateral area of the suprasellar region, the “parasuprasellar” area. Building on our detailed dissection, we introduce two novels endoscopic endonasal supraoptic (EESO) and endoscopic endonasal infraoptic (EEIO) approaches to access the parasuprasellar area. Indications and nuances of these approaches in treating 12 patients with tumors and aneurysms involving this area are also presented.

## Methods

### Anatomical Dissection

Five embalmed and injected adult cadaveric heads were used for endoscopic and microsurgical dissection. The anatomy research was approved by our institutional ethics committee. Endoscopic endonasal anatomical dissections were performed using rod lens endoscopes (4-mm diameter, 18-cm length, 0° and 30°, Karl Storz). An extended EEA to the sella, parasellar and suprasellar areas, involving wide sphenoidotomy, posterior ethmoidectomy, and posterior septectomy, followed by a transpterygoid approach, was performed for all cadaveric heads in a stepwise manner, as previously described (2, 3, 16). All intrasphenoidal landmarks were exposed, including the sella, tuberculum sellae, optic protuberances, carotid protuberances, medial opticocarotid recesses (MOCRs) and lateral opticocarotid recesses (LOCRs). The posterior ethmoidal artery (PEA) was also skeletonized.

**Figure 1:**
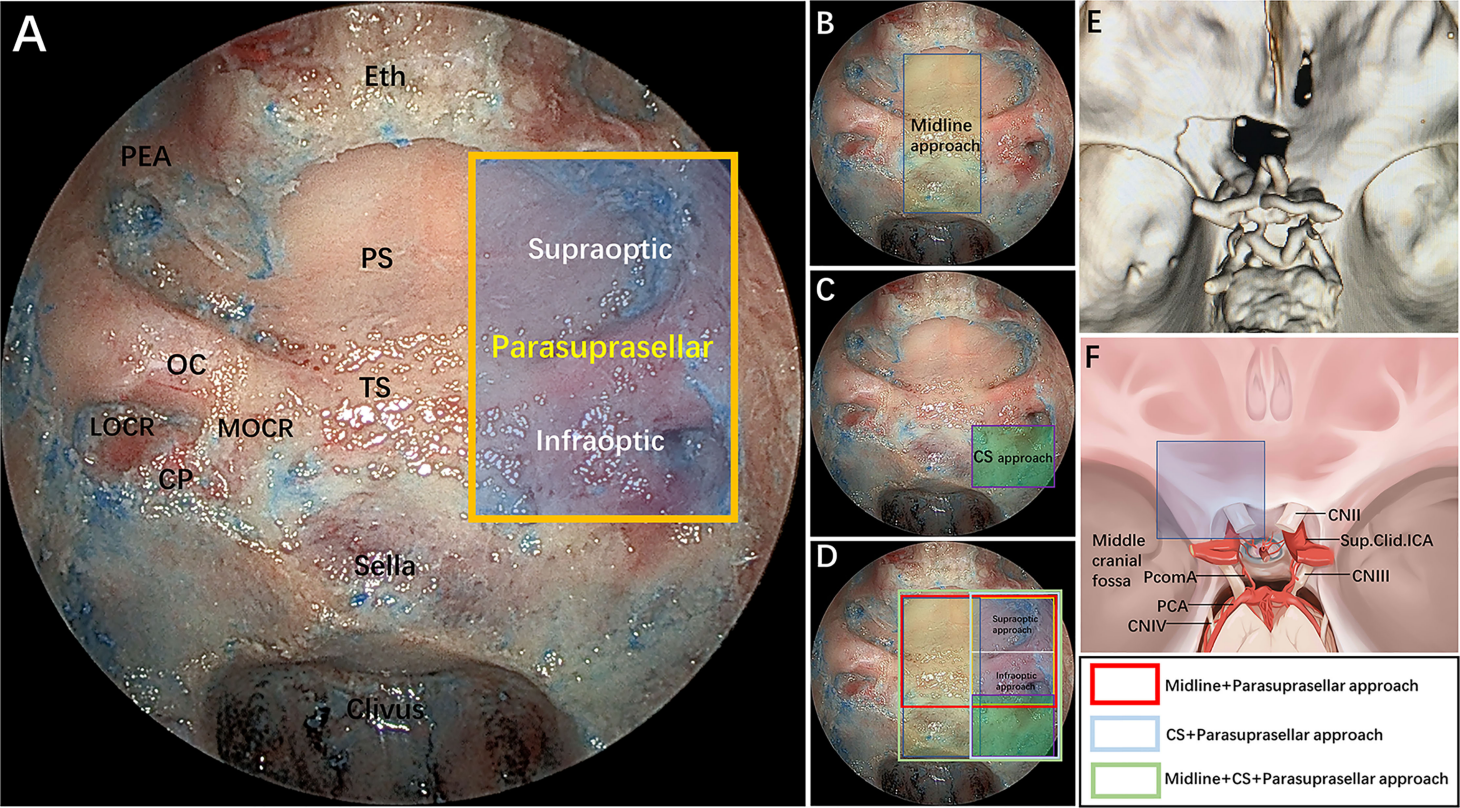
**(A)** Endoscopic endonasal view of our proposed parasuprasellar area and its surrounding essential anatomical structures. The parasuprasellar area is delineated by the yellow quadrangular space, which is limited medially by the medial edge of the MOCR, laterally by the lateral edge of the LOCR, superiorly by the PEA, and inferiorly by the inferior edge of the MOCR and LOCR. In addition, as bounded by the optic nerve, it can be divided into supraoptic and infraoptic regions. **(B–D)** Illustration showing different combinations of surgical modules in both midline (transsellar/transtuberculum/transplanum approach) and/or lateral (transcavernous and parasuprasellar approach) planes. Note that the parasuprasellar approach includes supraoptic and infraoptic approaches. The various combinations of endoscopic corridors are indicated with different quadrangular colors (figure in the lower right corner). Red marks the combination of the endoscopic endonasal midline approach and parasuprasellar approach; blue represents the combination of the transcavernous approach and parasuprasellar approach; green shows the combination of the midline approach, transcavernous approach and parasuprasellar approach. **(E)** Three-dimensional (3D) reconstruction of a postoperative fine-slice CT scan showing the extent of bone removal through the supraoptic and infraoptic approaches. **(F)** Artistic illustration demonstrating maximum bone removal (blue square) in the parasuprasellar area *via* the supraoptic and infraoptic approach. Eth, cribriform plate of the ethmoid; PEA, posterior ethmoidal artery; PS, planum sphenoidale; OC, optic canal; TS, tuberculum sellae; MOCR, medial optocarotid recess; LOCR, lateral optocarotid recess; CP, carotid protuberance; CS, cavernous sinus; CN II, optic nerve; CN III, oculomotor nerve; CN IV, trochlear nerve; PcomA, posterior communication artery; Sup.Clid. ICA, supraclinoidal internal carotid artery; PCA, posterior cerebral artery. The figure is available in color only online.

We defined the parasuprasellar area as a quadrangular space, and its main contents included the ON, the ICA and its proximal and distal dural rings, the ophthalmic artery (OA), and the ACP. The PEA is defined as the upper boundary of the parasuprasellar area. The inferior boundary is formed by the horizontal connection between the inferior edge of the LOCR and MOCR. The medial boundary is the vertical connection between the medial edge of the MOCR and the PEA, and the lateral boundary is the vertical connection between the lateral edge of the LOCR and the PEA. In addition, we divided the parasuprasellar area into 2 compartments based upon the ON: supraoptic and infraoptic compartments (**Figure 1A**). The EESO and EEIO approaches were performed to access the supraoptic and infraoptic regions, respectively.

Particular attention should be paid to the anatomy of the parasuprasellar area and its vicinity from an endoscopic perspective as well as to the stepwise surgical techniques related to the safe dissociation of the ON and OA. After completing the endonasal procedures, the extent of bone and dural removal from the parasuprasellar area was further evaluated from the intracranial superior view (**Figures 1E,F**). Several anatomical parameter measurements were also measured and recorded.

### Patient Population

From January 2016 to March 2020, we retrospectively reviewed 12 patients with lesions invading the parasuprasellar area and for whom the EESO and EEIO approaches were performed either alone or in combination. T1/T2WI and Gd-enhanced T1WI were performed in 10 patients with tumors, and contrast-enhanced postoperative MRI was performed as follow-up on postoperative day 1 and at 3 months after surgery. The remaining two patients with multiple aneurysms underwent pre- and postoperative cerebrovascular examinations, including CT angiography and digital subtraction angiography (DSA). All patients also underwent preoperative thin-slice CT scans to evaluate the extent of OC and ACP involvement. Preoperative BOT was performed to evaluate whether collateral circulation could be compensated; if poor, an endovascular stent or bypass would be prepared. Intraoperative electrophysiological monitoring, particularly visual evoked potentials (VEP), was used routinely throughout the procedure. Intraoperative neuronavigation and Doppler ultrasound were also applied to determine the exact course of the ICA. Additionally, the paraclival ICA was exposed in advance for proximal control. Special attention was given to the ophthalmological evaluation, including visual acuity and visual field, and limitations of ocular motility were observed by an ophthalmologist for all patients preoperatively and 3 to 6 months postoperatively. All medical records, including symptoms, neuroimaging, intraoperative videos, technical nuances, and surgical outcomes, were reviewed and analyzed retrospectively (**Table 1**).

**Table 1:** Summary characteristics and outcomes of all 12 clinical cases.

Case No.	Age/Sex	Size(cm)	Diagnosis	Preop		Other Symptoms	ICA, ACA, MCA Involvement	Previous Treatment	Surgical Approach	Anterior Clinoidectomy	EOR	Postop		Complications	Follow-Up(months)
				Visual Acuity	Visual Field							Visual Acuity	Visual Field		
**1**	56/F	2.1×1.9×1.7	Lt ACP meningioma	Visual loss (lt)	Upper hemianopsia	Dizzy	No	None	TPA+SOA	No	GTR	Improvement	Normal	None	56
**2**	25/F	2.5×1.9×1.4	Rt ACP meningioma	Visual loss (rt)	Rt temporal hemianopsia	Headache	Yes, all attached	Pterional approach	TMA*+IOA	Yes	GTR	Worse (rt), unchanged (lt)	Rt temporal hemianopsia	None	50
**3**	56/F	2.1×1.8×1.7	Lt ACP meningioma	Normal	Normal	Dizzy	Yes, all attached	None	TMA+SOA+IOA	No	GTR	Stable	Normal	None	36
**4**	37/F	3.0×1.7×1.4	Lt ACP meningioma	Visual loss (lt)	Lt temporal hemianopsia	None	Yes, all encased	Pterional approach	TMA+TPA+TCA +SOA+IOA	Yes	STR	Stable	Lt temporal hemianopsia	CNIII pasly	45
**5**	12/M	2.8×3.1×3.9	Osteogenic meningioma	Visual loss	Lt amaurosis	None	No	None	TMA+TPA+SOA +IOA	Yes	GTR	Marked improvement	Lt temporal hemianopsia	Transient DI	18
**6**	45/F	3.7×4.0×3.9	Pituitary adenoma	Visual loss	Lt temporal hemianopsia	Headache	Yes, ICA and ACA encased	None	TMA+TPA+TCA +SOA+IOA	No	GTR	Stable	Lt temporal hemianopsia	CSF leak	30
**7**	52/F	3.5×2.7×2.9	Pituitary adenoma	Visual loss	Rt temporal hemianopsia	None	Yes, ICA encased, ACA and MCA attached	Endonasal endoscopic approach	TMA+TPA+TCA +SOA+IOA	Yes	GTR	Improvement	Normal	Panhypopituitarism Transient DI	22
**8**	56/M	4.5×2.7×3.0	Pituitary adenoma	Visual loss (rt)	Bitemporal hemianopsia	Headache, hypomnesis	Yes, ICA encased, ACA attached	Endonasal microscope approach	TMA+TPA+TCA +SOA+IOA	No	GTR	Marked Improvement	Normal	None	29
**9**	47/F	lt paraclinoid: 0.7×0.8 lt ophthalmic: 0.4×0.3 It cav-ICA: 0.5×0.4	Multiple aneurysms: lt paraclinoid, lt ophthalmic, lt cav-ICA	Normal	Normal	Headache	NA	None	TMA+TPA+IOA	Yes	Clipping of lt paraclinoid, lt ophthalmic aneurysms	Stable	Normal	None	58
**10**	62/F	lt paraclinoid: 0.75×0.42 rt paraclinoid:2.26×2.17 Acom: 0.4×0.3	Multiple aneurysms: lt paraclinoid, rt paraclinoid, Acom	Visual loss	Normal	Dizzy	NA	None	TMA+IOA	No	Clipping of lt paraclinoid, Acom aneurysms	Marked improvement	Normal	None	44
**11**	30/M	2.3×2.2×1.9	Craniopharyngioma	Visual loss(rt)	Lower marginal field	None	Yes, all encased	Subfrontal approach, V-P shunting	TMA+SOA+IOA	No	GTR	Improvement	Rt hemianopsia	Hypothyroidism DI	26
**12**	37/F	1.8×1.4×1.0	Meningeal IgG4- related disease	Visual loss(rt)	Rt temporal hemianopsia	None	No	None	TMA+SOA+IOA	Yes	GTR	Improvement	Normal	CNIII pasly	52

ICA, internal carotid artery; ACA, anterior cerebral artery; MCA, middle cerebral artery; EOR, extent of resection; ACP, anterior clinoid process; TPA, transpterygoid approach; TMA, transmidline approach;TCA, transcavernous approach; SOA, supraoptic approach; IOA, infraoptic approach; GTR, gross-total resection; STR, subtotal resection; DI, diabetes insipidus; NA, not applicable; Acom, anterior communicating;Cav-ICA=cavernous segment of ICA; * including endoscopic endonasal transsellar, transtuberculum, transplanumh and transclivus approaches.

## Results

### EESO Approach to the Supraoptic Region

#### Stage 1: Recognition and Exposure of the Supraoptic Recess

**Figure 2:**
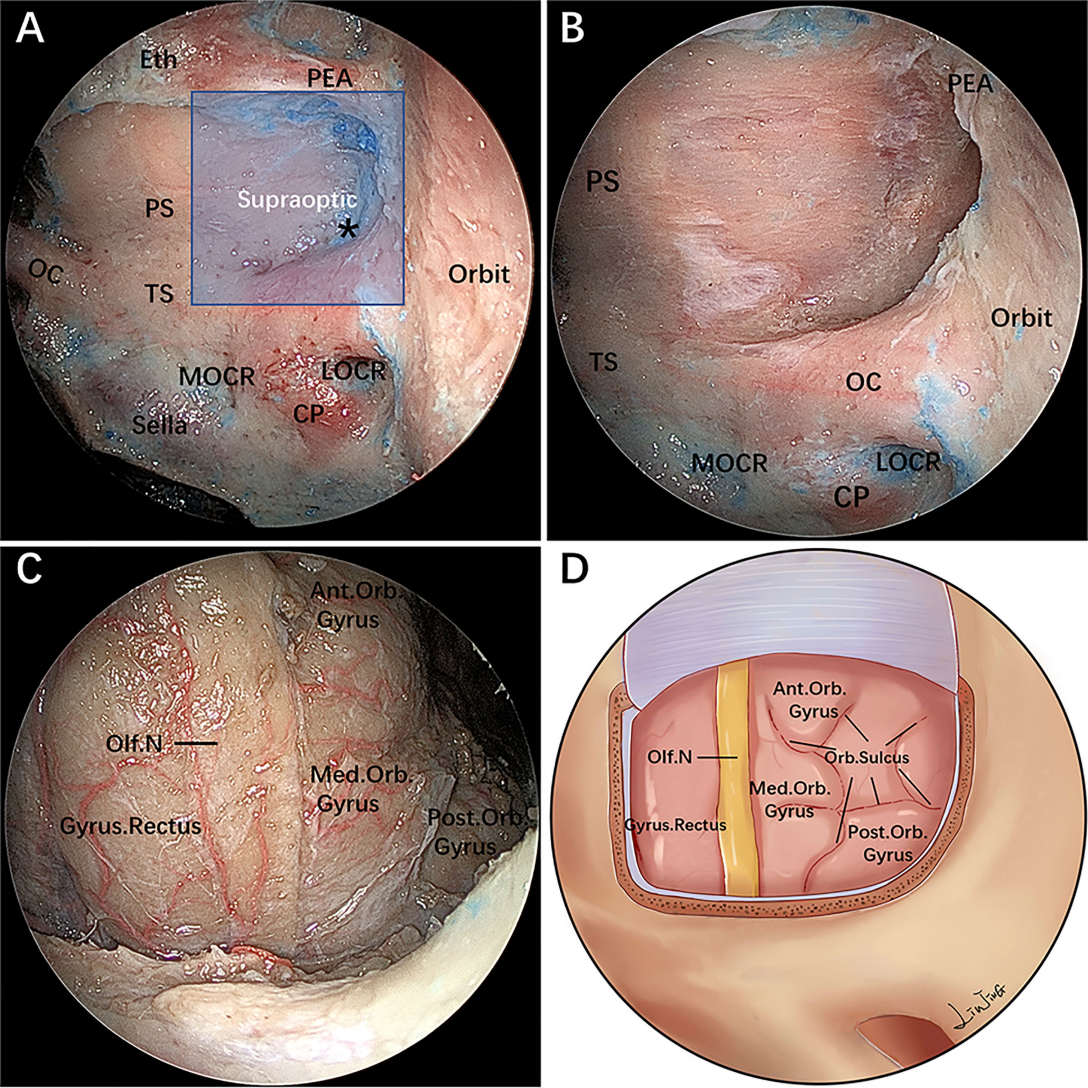
Endoscopic endonasal supraoptic (EESO) approach: stepwise dissection to the supraoptic region in a colored silicone-injected cadaveric specimen. **(A)** Panoramic view of the sphenoid sinus floor with its anatomical landmarks. The blue quadrangular zone marks the supraoptic region, and the black asterisk represents the supraoptic recess. **(B)** A closer view of the supraoptic region after removal of the medial portion of the lesser sphenoid wing and OC unroofing. **(C)** After removing the bone and dura mater lateral to the planum sphenoidale, the gyrus rectus, olfactory nerve, medial orbital gyrus, anterior orbital gyrus and post orbital gyrus of the frontal lobes were exposed, viewed with a 0° endoscope. **(D)** Artistic illustration demonstrating the contents of the supraoptic region that can be reached *via* the EESO approach. Olf. N, olfactory nerve; Med.Orb. Gyrus, medial orbital gyrus; Ant.Orb. Gyrus, anterior orbital gyrus; Post.Orb. Gyrus, post orbital gyrus. The figure is available in color only online.

After undergoing an initial extended EEA, the PEA located in its osseous canal is identified and serves as the upper boundary of the supraoptic region; the artery can be ligated and transected to facilitate lateral mobilization. The supraoptic recess is pyramid-shaped, with its base abutting the sphenoid sinus and apex corresponding intracranially to the body of the lesser sphenoid wing (**Figure 2A**) (11). The supraoptic recess needs to be identified and sufficiently delineated because its removal is one of the key steps to achieve further lateral extension over the planum sphenoidale.

#### Stage 2: Removal of the Medial Portion of the Lesser Sphenoid Wing and OC Unroofing

During this stage, the base of the supraoptic recess is drilled with a small diamond burr, proceeding deeply in a medial-to-lateral direction toward the body of the lesser sphenoid wing. As a consequence, the most medial portion of the lesser sphenoid wing overlying the orbit is exposed and progressively drilled out. However, removal of the lateral portion of the lesser sphenoid wing is limited inferiorly by the intracanalicular portion of the ON and the superior aspect of the medial orbital walls, which serves as the main anatomical landmark of the superolateral boundary of this exposure (**Figure 2B**). Afterward, the roof wall of the OC is drilled.

#### Stage 3: Resection of the Lateral Dura of the Planum Sphenoidale and Exposure of the Orbital Gyrus

The dura mater of the planum sphenoidale is opened in a posterior-to-anterior direction, after which the gyrus rectus of the frontal lobe and the olfactory nerve are visible at this level. The residual lateral part of the dura mater of the planum sphenoidale can be safely removed using an outward-facing Kerrison rongeur. This maneuver permits visualization of the medial orbital gyrus, anterior orbital gyrus and post orbital gyrus of the frontal lobe (**Figures 2C,D**). At this time, the distance from the lateral edge of the olfactory nerve to the outermost edge of the orbital gyrus is measured with a ruler (**Table 2**).

**Table 2:** Relevant measurements.

Measurement	Mean ± SD, mm (right, n=5)	Mean ± SD, mm (left, n=5)	Mean ± SD, mm (total, n=10)
The length of the three sides of the ACP triangle:			
superomedial to superolateral vertices	4.30 ± 0.57	4.40 ± 0.65	4.35 ± 0.58
superomedial to inferior vertices	4.10 ± 0.55	4.20 ± 0.45	4.15 ± 0.47
superolateral to inferior vertices	3.80 ± 0.27	3.90 ± 0.42	3.85 ± 0.34
The lateral edge of the olfactory nerve to the outermost edge of the orbital gyrus	10.40 ± 0.65	10.30 ± 0.84	10.35 ± 0.71

### EEIO Approach to the Infraoptic Region

#### Stage 1: Removal of the Anterior Wall of the OC and Exposure of the Intracranial ON

**Figure 3:**
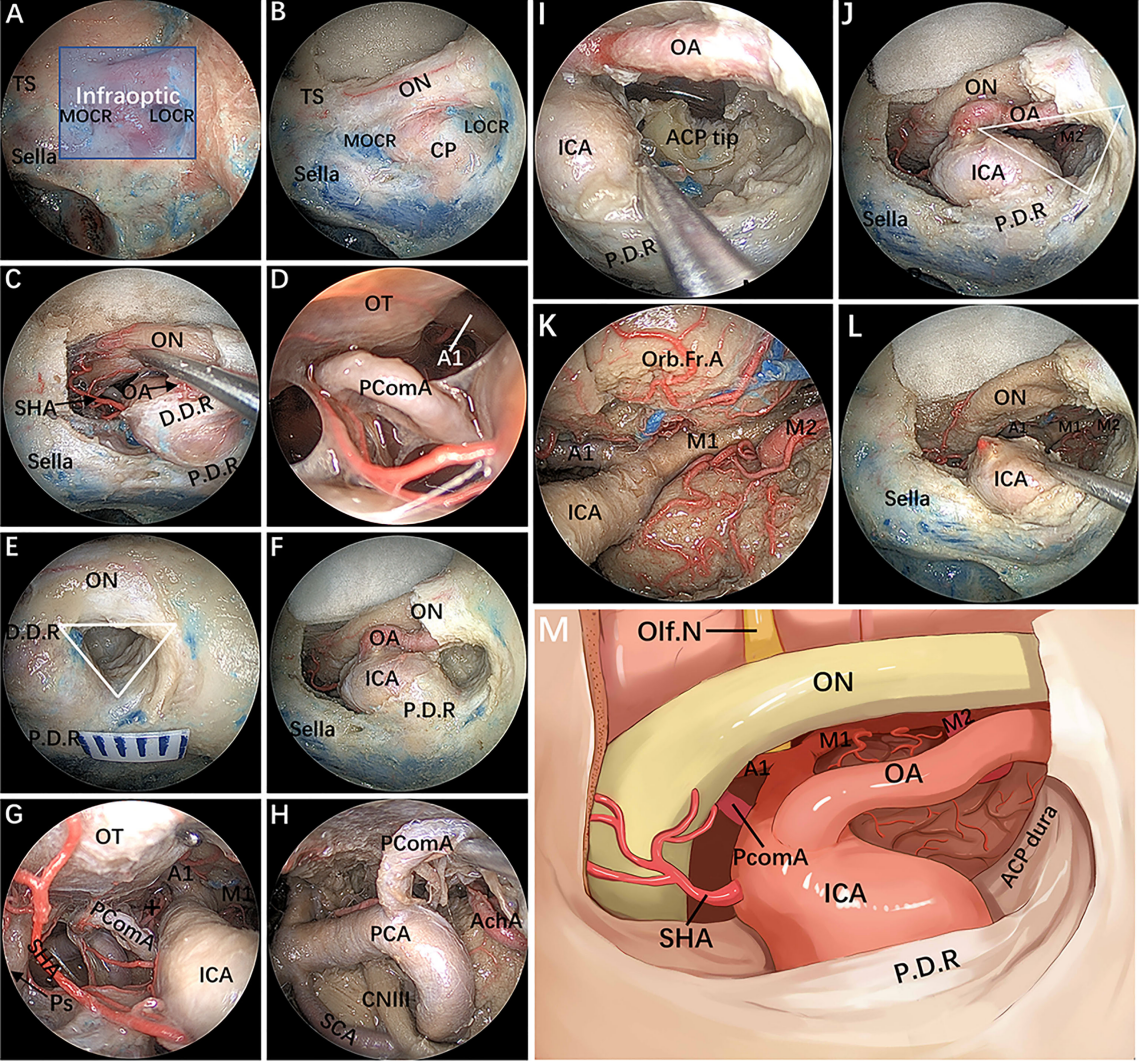
Endoscopic endonasal infraraoptic (EEIO) approach: stepwise dissection to the infraoptic region in a colored silicone-injected cadaveric specimen. **(A)** The main anatomical landmarks in the infraoptic region (blue zone) are shown. **(B)** Removal of the anterior wall of the OC in a medial-lateral direction up the orbit apex. **(C)** The dura overlying the intracranial ON was incised longitudinally to expose the origin of the OA; the diaphragm was incised toward the medial part of the DDR, and SHA and its branches were exposed. **(D)** A closer view shows more subdural contents, including the PcomA, OT and A1 segments of the anterior cerebral artery. **(E)** Drilling of the optic strut and showing the ACP triangle. **(F)** The DDR and ON sheath are opened to further safely dissociate the OA and ON. **(G, H)** The subdural neurovascular structures were explored again by gently lifting of the ipsilateral ON. The main structures are identified, including the PcomA, pituitary stalk, AchA and its branches (black plus sign) into the anterior perforating substance in the crural cistern, the CNIII passing between the PCA and SCA into the cavernous sinus, and the bifurcation of the ICA. **(I)** The base and tip of the ACP can be further removed by gentle lifting of the OA or medial mobilization of the paraclinoidal ICA. **(J)** The ACP triangular is further enlarged. **(K)** The sylvian cistern was visible, and the ICA bifurcation was exposed between the frontal and temporal lobes; more laterally, the middle cerebral artery (MCA) bifurcation was observed at the level of its insular portion. **(L)** The OA was transected, and the operation space of the EEIO corridor was further enlarged. **(M)** Artistic illustration showing the main contents of the infraoptic region that can be reached *via* the EEIO approach. Note the ON has been slightly elevated. ON = optic nerve; OA = ophthalmic artery; SHA = superior hypophyseal artery; D.D.R, distal dural ring; P.D.R, proximal dural ring; ICA, internal carotid artery; OT, optic tract; AchA, anterior choroidal artery; SCA, superior cerebellar artery; Orb.Fr.A, orbital frontal artery; ACP, anterior clinoid process; M1, sphenoidal segment of the middle cerebral artery; M2, insular segment of the middle cerebral artery. The figure is available in color only online.

After the bone of the anterior wall of the OC is removed in a medial-lateral direction up to the orbital apex, the dura overlying the intracranial ON is incised longitudinally to expose the origin of the OA (**Figures 3A–C**). Of note, the most common relationship of the origin of the OA to the intracranial ON is an inferomedial location; thus, opening the dura through a cut parallel to the ON in its upper half reduces the risk of damaging the artery (17, 18). To gain working space in the medial region of the paraclinoidal ICA, the diaphragm is incised toward the medial part of the distal dural ring (DDR). Following this, intradural exploration of the main neurovascular structures is performed. The pituitary stalk, superior hypophyseal artery (SHA) and its branches are exposed by gently lifting the ipsilateral ON (**Figure 3C**). Sliding deeper, the posterior communicating artery (PcomA) and the A1 segment of the anterior cerebral artery are identified using the space between the SHA and the ON (**Figure 3D**).

#### Stage 2: Drilling of the Optic Strut and Dissociation of the OA

Surgery in the lateral compartment of the paraclinoidal ICA requires removal of the optic strut, which corresponds to the LOCR from the endonasal perspective (19). Three vertices of this recess (resembling a triangle in shape) are identified: the superomedial, superolateral, and inferior vertices. The distance between each vertex of the LOCR is measured (**Table 2**). With regard to safe removal of the three vertices in turn to the base of the ACP, we have specifically discussed the relevant anatomical details and surgical nuances in a previous publication (20). Once drilling of the optic strut is concluded (**Figure 3E**), the DDR is opened to further safely dissociate the OA. Next, proper exposure of the ON and OA surrounded by the dural sheath is performed. At this stage, the courses of the precanalicular ON and OA are entirely exposed (**Figure 3F**). This maneuver aims to separate the ON from the OA, thus widening the surgical corridor of the infraoptic approach. The main structures are identified, including the PcomA, the anterior choroidal artery and its branches into the anterior perforating substance, the oculomotor nerve passing between the posterior cerebral artery and superior cerebellar artery into the CS, and the bifurcation of the ICA (**Figures 3G, H**).

#### Stage 3: Removal of the ACP and Severing of the OA

The ACP is now only connected to part of the lesser sphenoid wing. By using this ACP triangle space and following gentle medial mobilization of the paraclinoidal ICA, drilling and curettage of the base and tip of the ACP becomes feasible (**Figures 3I,J**). However, this step is performed with extreme care to avoid injury to the ICA, OA, ON and oculomotor nerve, and tailored bone drilling is strongly suggested according to disease-specific management. Once removal of the ACP tip is achieved, the dura of the inferior surface of the ACP is incised with angled scissors or coagulated. At this moment, the sylvian cistern is visible, and M1 is exposed between the frontal and temporal lobes; more laterally, the middle cerebral artery bifurcation is observed at the level of its insular portion (**Figure 3K**). Some perforating vessels from M1 are also identified. Finally, the OA is transected, and the operating space of the infraoptic approach is further enlarged (**Figures 3L,M**).

### Combined EESO and EEIO Approaches for the Parasuprasellar Area

**Figure 4:**
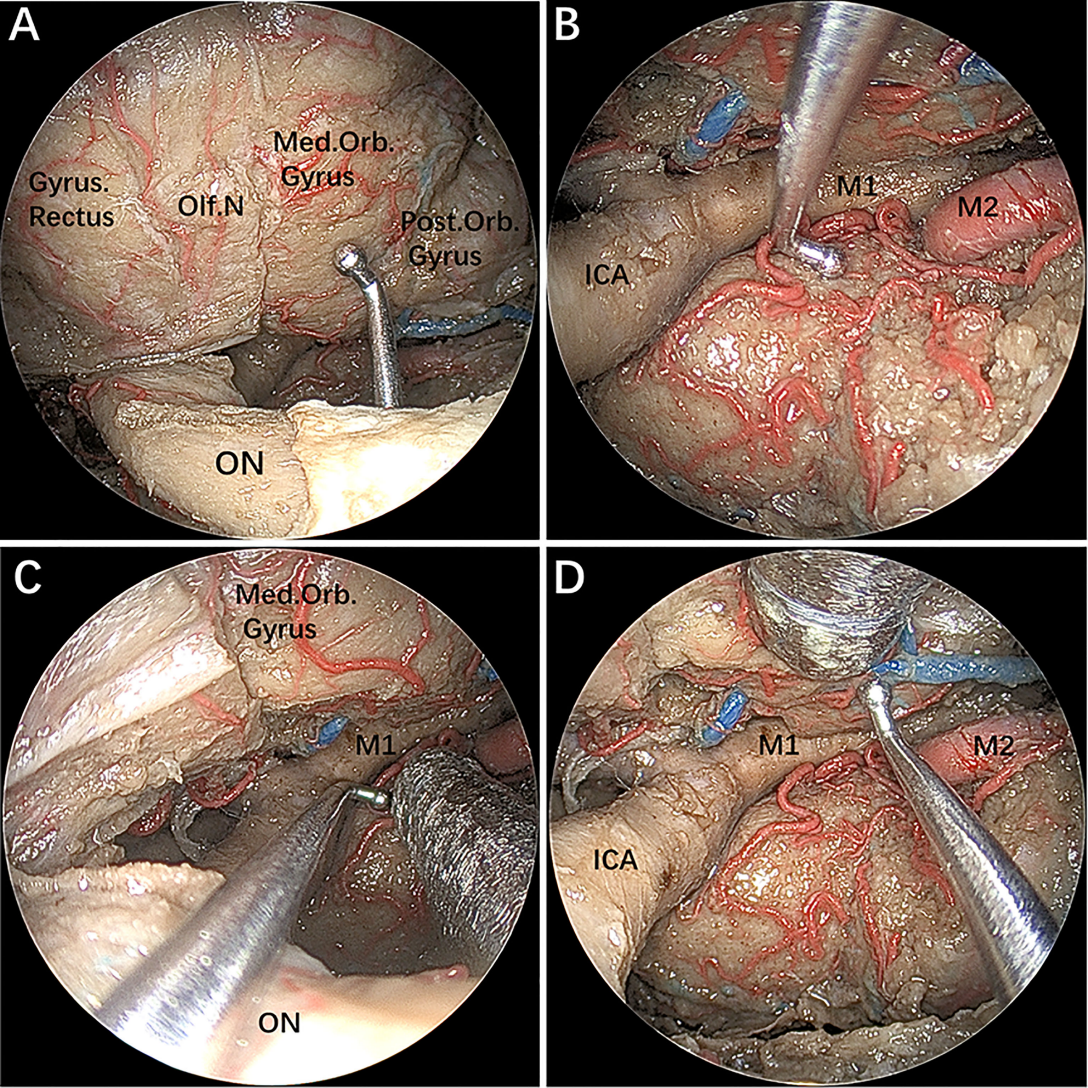
**(A–D)** The instruments demonstrating a combined supraoptic and infraoptic approach to access the parasuprasellar area. The figure is available in color only online.

After completing the above step-by-step dissection, combined EESO and EEIO approaches for accessing the parasuprasellar area were performed, as shown in **Figure 4**. It should be emphasized that the EESO and EEIO approaches can be used either alone or in combination; they can also be combined with the endoscopic endonasal midline approach (transsellar/transtuberculum/transplanum) and transcavernous approach according to the disease-specific location (**Figures 1B–D**).

### EESO and EEIO Approaches: Case Series

The EESO and EEIO approaches were successfully performed either alone or in combination for 12 patients harboring tumors and aneurysms involving the parasuprasellar area (**Table 1**). The mean patient age was 42.9 years (range 12–62 years); there were 3 males and 9 females. The most common presenting symptom was visual deficit, including visual loss and visual field defects. Other symptoms were headache, dizziness, and hypomnesis. Three patients had previously undergone TCA, and 2 patients underwent an endonasal (microscope or endoscopic) approach at other institutions. The final diagnoses were meningiomas in 5 patients, pituitary adenoma in 3 patients, multiple aneurysms in 2 patients, and meningeal IgG4-related disease and craniopharyngioma in 1 patient each. Gross total tumor resection was achieved in 9 patients; subtotal resection was achieved in 1 patient. There were 2 patients with multiple aneurysms. One case of anterior communication and paraclinoid aneurysms were clipped *via* a pure EEIO approach; a contralateral giant paraclinoid aneurysm was secondarily embolized at 2 months after the operation. The other patient harbored ophthalmic and paraclinoid aneurysms that were also clipped through the EEIO approach, although an intracavernous aneurysm was left untreated due to its location and size. Postoperative visual acuity improved in 7 patients, remained unchanged in 4 patients, and deteriorated in 1 patient in the right eye. The postoperative visual field was normal in 7 patients, whereas 5 still had unilateral temporal hemianopsia. Two patients experienced transient diabetes insipidus (DI), and 1 patient developed postoperative panhypopituitarism, which normalized by the 3-month follow-up. One patient experienced permanent DI and hypothyroidism, and postoperative hormone replacement therapy was required in the follow-up period. Postoperative cerebrospinal fluid (CSF) leakage occurred in 1 patient, and endoscopic endonasal repair was performed. Postoperative oculomotor nerve palsy developed in 2 patients; fortunately, it resolved completely in one patient by the 1-month follow-up, and significantly improved in the other patient by the 6-month follow-up. No postoperative intracranial infection or ICA injury occurred in this series.

## Discussion

Skull base pathologies encompassing the suprasellar lateral area, including the lateral aspect of the planum sphenoidale and the tight junction region of the OC, ACP, and ICA and its dural rings, still pose unique surgical challenges for neurosurgeons in terms of subsequent morbidity and gross total resection (10, 12, 20–22). These pathologies typically involve intra- and extracranially, tend to displace the ON from above and/or below, erode osseous and dural structures, and even involve the ICA bifurcation. Hence, TCAs for complete resection of these lesions have a high potential morbidity, even for skilled and experienced neurosurgeons. Today, endoscopy, which offers a wider, close-up view of the surgical field, is used broadly in skull base surgery. Although it has the disadvantage of increasing the rate of CSF leakage, the potential advantages of the EEA compared to conventional TCAs include avoiding brain retraction, improved visualization, better protection of surrounding neurovascular structures, and shorter hospital stay (1–3, 23–25). These advantages are similar when comparing the EEA and different TCAs for lesions involving the lateral area of the suprasellar region. The EEA not only provides the most straightforward surgical route parallel to the growth axis of the tumor but also, most importantly, allows for better control of the paraclinoidal ICA, which constitutes a lateral barrier to directly approaching these regions through the sphenoid sinus. However, the intricate anatomical complexity and lack of anatomical detail suitable for surgical exploration make these regions among the most challenging areas to approach.

In this paper, we describe the surgical anatomy of the lateral area of the suprasellar region, termed the “parasuprasellar” area, from the endoscopic perspective. Moreover, we introduce the EESO and EEIO approaches to access this complex area. The same techniques were applied in 12 consecutive patients harboring tumors and aneurysms involving the parasuprasellar area. To the best of our knowledge, this is the first report on the EESO and EEIO approaches.

### Approach Selection and Technical Considerations

**Figure 5:**
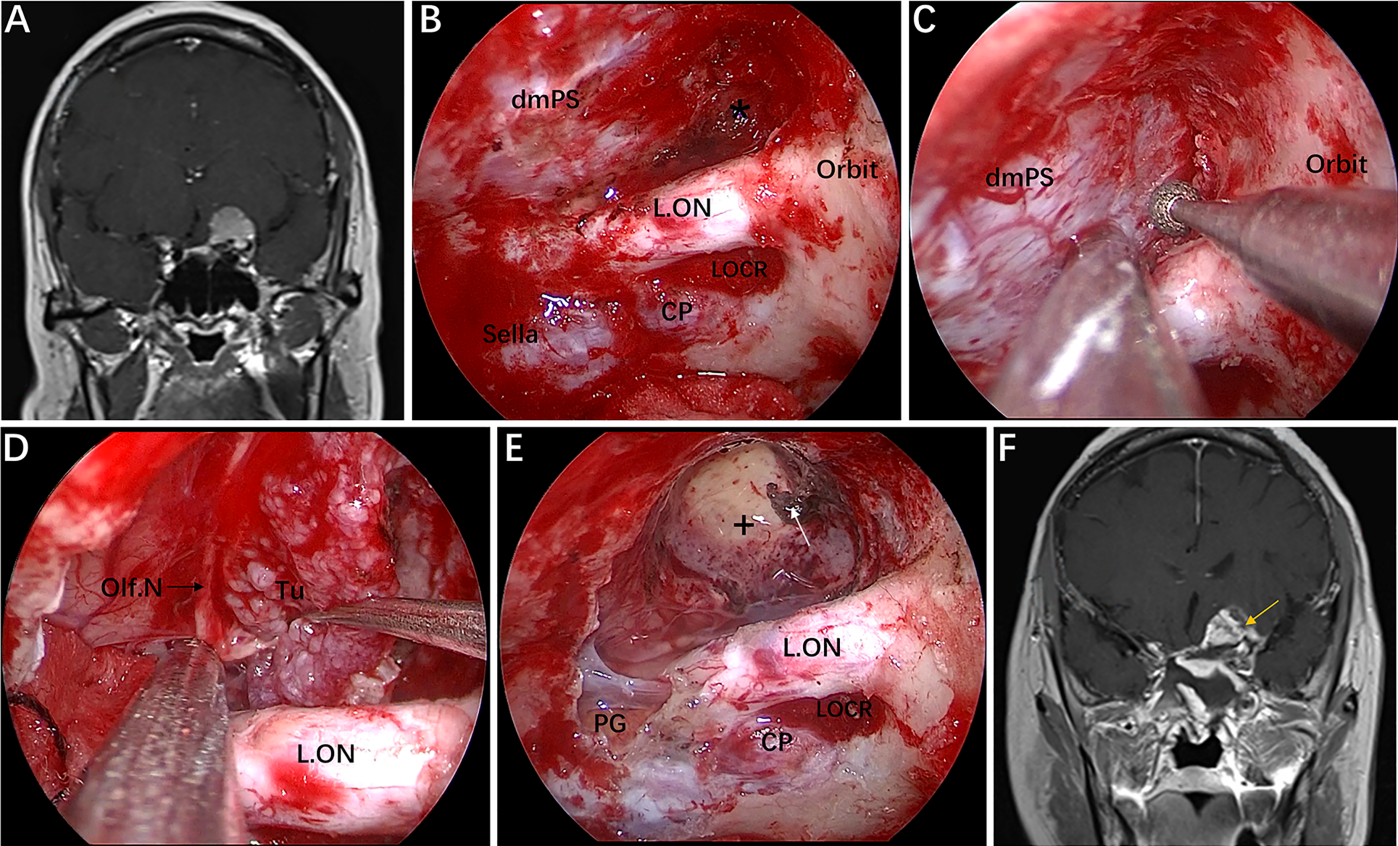
Case 1, a 56-year-old woman, presented with a 1-year history of dizziness and progressive left visual loss for more than 1 month. **(A)** Preoperative coronal Gd-enhanced MRI showing a left ACP meningioma. **(B–E)** Intraoperative images. **(B)** Exposure of the lateral dura of the planum sphenoidale and supraoptic recess (black asterisk). **(C)** Drilling the roof wall of the optic canal and supraoptic recess in a medial-to-lateral direction toward the body of the lesser sphenoid wing. **(D)** Separation of the tumor from the olfactory nerve through the supraoptic approach. **(E)** Final endoscopic view after complete tumor removal. The white arrow represents the severed posterior ethmoidal artery. **(F)** Postoperative coronal MRI showing total tumor removal. The yellow arrow represents the autologous fat used during the operation. + = tumor cavity. The figure is available in color only online.

**Figure 6:**
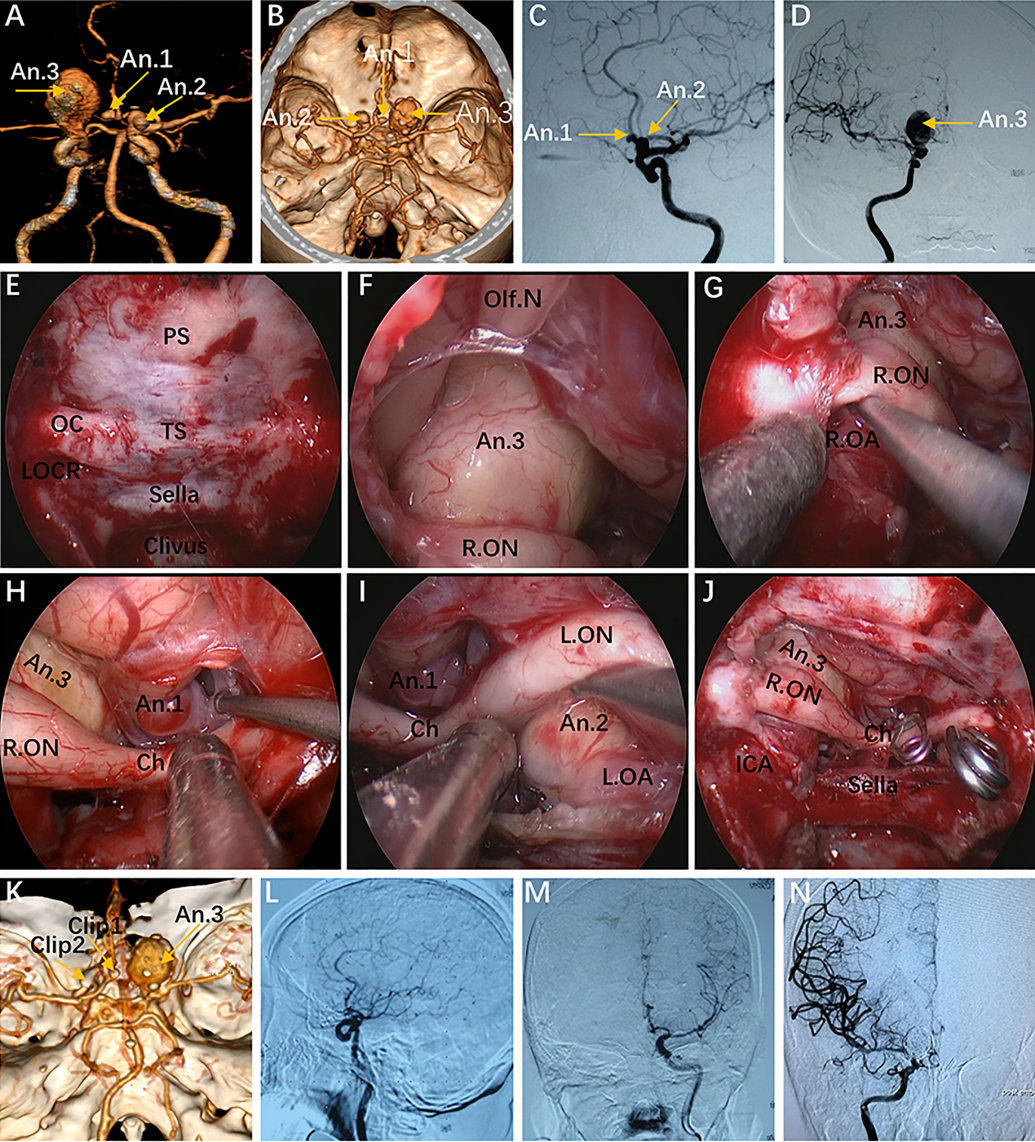
Case 10, a 62-year-old woman, presented with visual loss for 2 months. **(A, B)** Preoperative CT angiography **(A)** and a 3-D reconstruction image **(B)** showing an Acom aneurysm (An1), a left praclinoid aneurysm (An2), and a large right paraclinoid aneurysm (An3). **(C, D)** Anteroposterior view **(C)** and lateral view **(D)** of bilateral internal carotid artery (ICA) injection of digital subtraction angiography (DSA) confirming an Acom aneurysm, a left praclinoid aneurysm, and a large right paraclinoid aneurysm. **(E–J)** Intraoperative images. **(E)** Endoscopic view of the skull base showing important bony landmarks. **(F, G)** Intraoperative image demonstrating an aneurysm body with a thrombus above the right optic nerve that cannot be clipped through an endonasal approach. **(H, I)** Exposure to the Acom aneurysm **(H)** and left paraclinoid aneurysm **(I)**. **(J)** Intraoperative picture showing that both aneurysms were clipped successfully after proximal control. **(K)** Postoperative CT angiography confirmed the patency of the bilateral A2 and distal ICA. **(L, M)** Postoperative lateral view **(L)** and anteroposterior view **(M)** of left ICA injection of DSA, revealing complete obliteration of An1 and An2. **(N)** Postoperative anteroposterior view of right ICA injection of DSA, showing complete obliteration of the An3 after second-stage coiling. An, aneurysm; R.OA, right ophthalmic artery; L.OA, left ophthalmic artery; Ch, optic chiasm. The figure is available in color only online.

**Figure 7:**
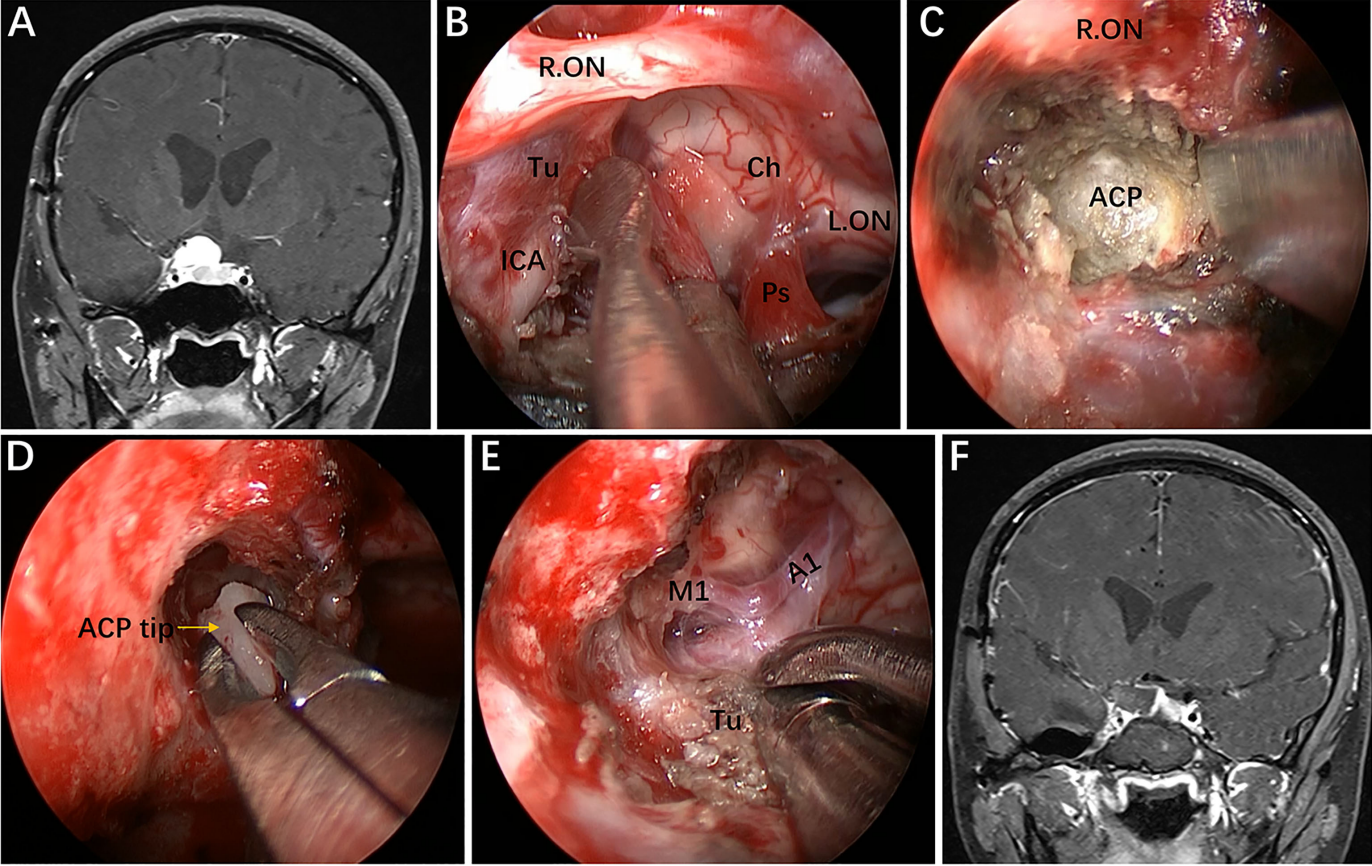
Case 2, A 25-year-old woman presented with headache and decreased vision in her right eye for 6 months. **(A)** Preoperative coronal Gd-enhanced MRI demonstrating a right recurrent ACP meningioma. **(B)** Removal of the tumor on the medial and upper parts of the supraclinoidal ICA. **(C, D)** Removal of the ACP invaded by the tumor. **(E)** Separation of tumor adhering to the bifurcation of the ICA through the infraoptic corridor. **(F)** Postoperative MRI showing total tumor removal. Tu = tumor; Ps = pituitary stalk; ACP = anterior clinoid process. Some panels **(7A, D, F**) of the figure have been published in *Journal of Neurosurge*ry. Published with permission. The figure is available in color only online.

**Figure 8:**
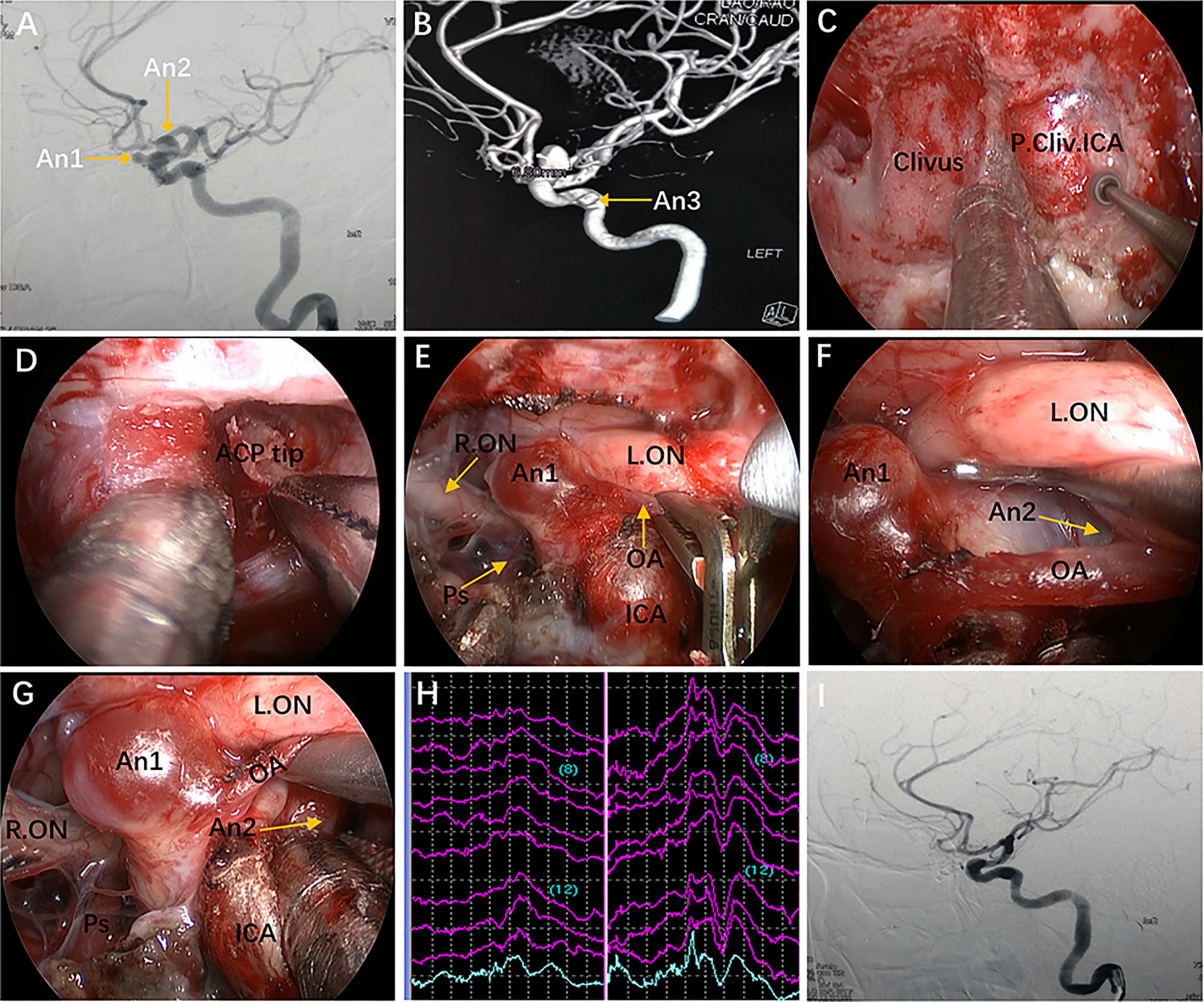
Case 9, a 47-year-old woman, presented with headache for 3 months. **(A)** Preoperative lateral view of left internal carotid artery (ICA) injection for digital subtraction angiography (DSA) showing an ophthalmic aneurysm (An1) and a paraclinoid aneurysm (An2). **(B)** anteroposterior view of the 3D reconstruction images showing another aneurysm (An3) located in the cavernous segment of the ICA. C-G: Intraoperative images. **(C)** Exposure of the paraclival ICA for proximal control in advance. **(D)** Removal of the ACP tip. **(E)** Temporary occlusion of the OA. **(F)** Exposure of the An2 neck by lifting the ipsilateral optic nerve. **(G)** Clip application to the An2 neck after proximal control. **(H)** Intraoperative visual evoked potential monitoring changed after temporary occlusion of the OA. **(I)** Postoperative lateral view of DSA showing complete obliteration of An1 and An2. Some panels **(8A, B, G, I)** of the figure have been published in *Journal of Neurosurgery*. Published with permission. The figure is available in color only online.

**Figure 9:**
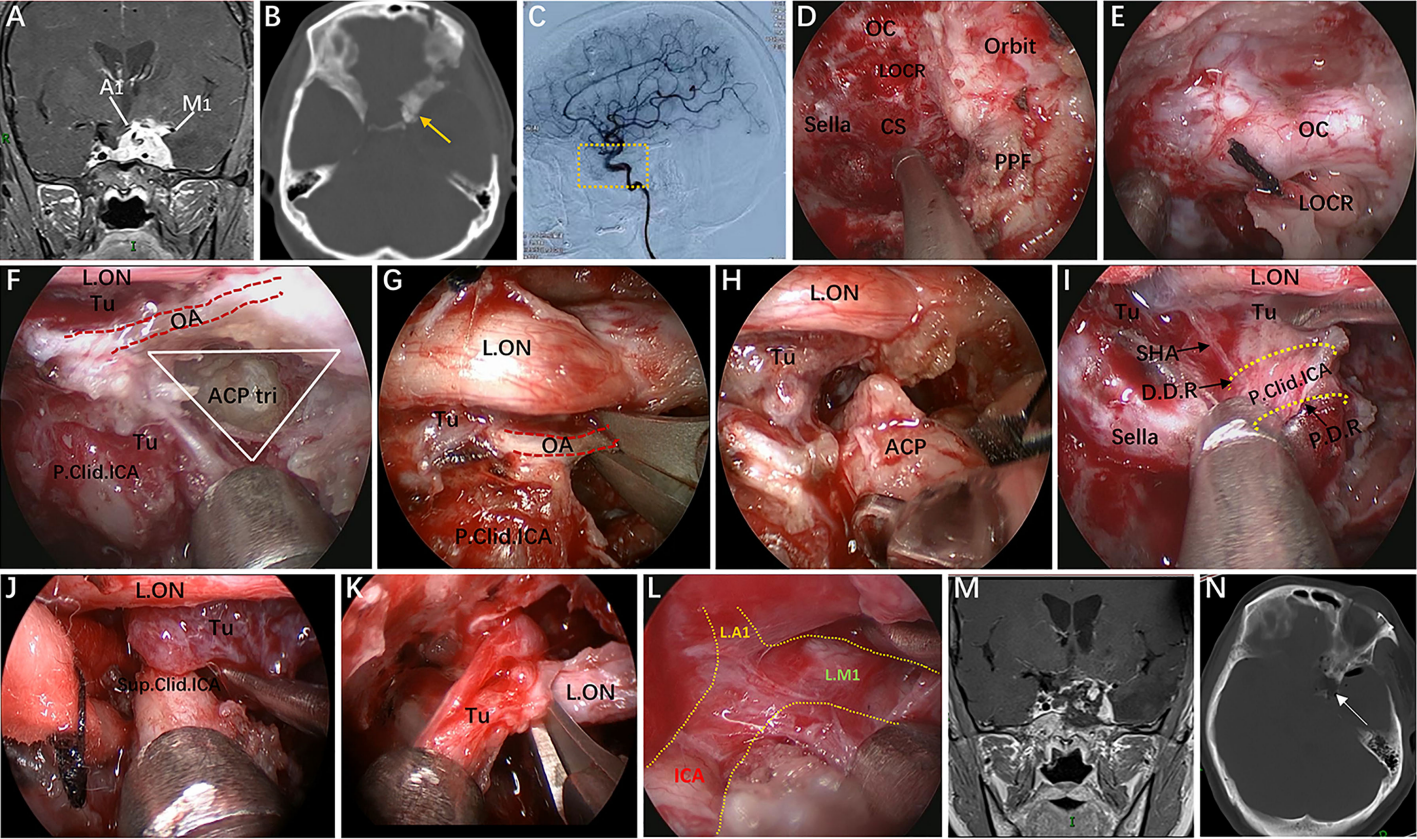
Case 4, a 37-year-old woman, presented with progressive visual loss in the left eye for 2 years. **(A)** Preoperative coronal Gd-enhanced MRI showing a left recurrent ACP meningioma involving the right cavernous sinus and encasement of the ICA and its bifurcation. **(B)** Preoperative CT scans. The yellow arrow indicates the hyperplastic ACP. **(C)** Preoperative lateral view of left ICA injection of digital subtraction angiography (DSA) showing that the ophthalmic artery was not visible. **(D–L)** Intraoperative images. **(D)** Endoscopic view of important bony landmarks after the initial transpterygoid approach. **(E)** Exposure of the thickened OC and drilling of its roof and anterior walls. **(F)** Endoscopic exposure of the ACP triangle. The red dotted line indicates the course of the imaginary occluded OA. **(G)** Sacrifice of the OA surrounded by tumors and involving the dura. **(H)** Removal of the hyperplastic ACP. **(I–K)** Resection of the tumor on the medial and lateral sides of the supraclinoidal ICA through the combined supraoptic and infraoptic approaches. **(L)** Endoscopic exposure of the bifurcation of the ICA covered by arachnoid membrane. **(M)** Corresponding postoperative coronal MRI showing subtotal tumor resection, with part of the tumor remaining in the lateral wall of the left CS. **(N)** Postoperative CT scan showing ACP resection. The white arrow represents the removed ACP. Some panels **(9A, B, M, N)** of the figure have been published in *Journal of Neurosurgery*. Published with permission. The figure is available in color only online.

**Figure 10:**
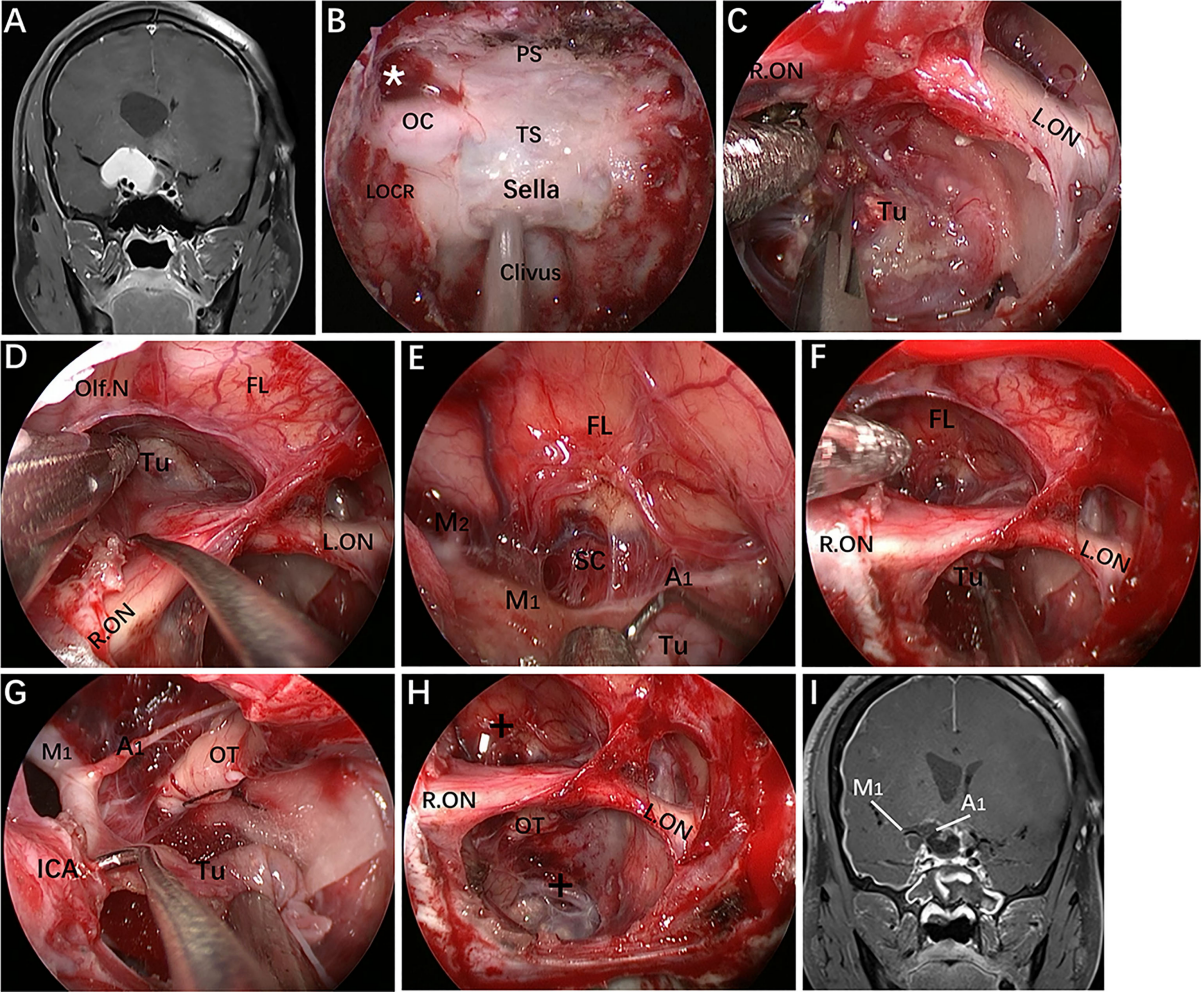
Case 11, a 30-year-old man, presented with progressive loss of vision for 6 months. **(A)** Preoperative coronal Gd-enhanced MRI demonstrating a recurrent intra- and suprasellar craniopharyngioma with right sylvian fissure extension. **(B–H)** Intraoperative images. **(B)** Endoscopic view of the skull base showing important bony landmarks. The white asterisk marks the supraoptic recess. **(C)** Removal of intrasellar tumor. **(D)** Extracapsular separation of the tumor adhering to the frontal lobe through the supraoptic corridor. **(E)** Endoscopic view of the sylvian fissure during tumor removal. **(F, G)** Sharp dissection of the tumor away from the neurovascular structures by the infraoptic corridor. **(H)** Final endoscopic view after complete tumor removal. **(I)** Postoperative coronal MRI showing total tumor removal. The figure is available in color only online.

**Figure 11:**
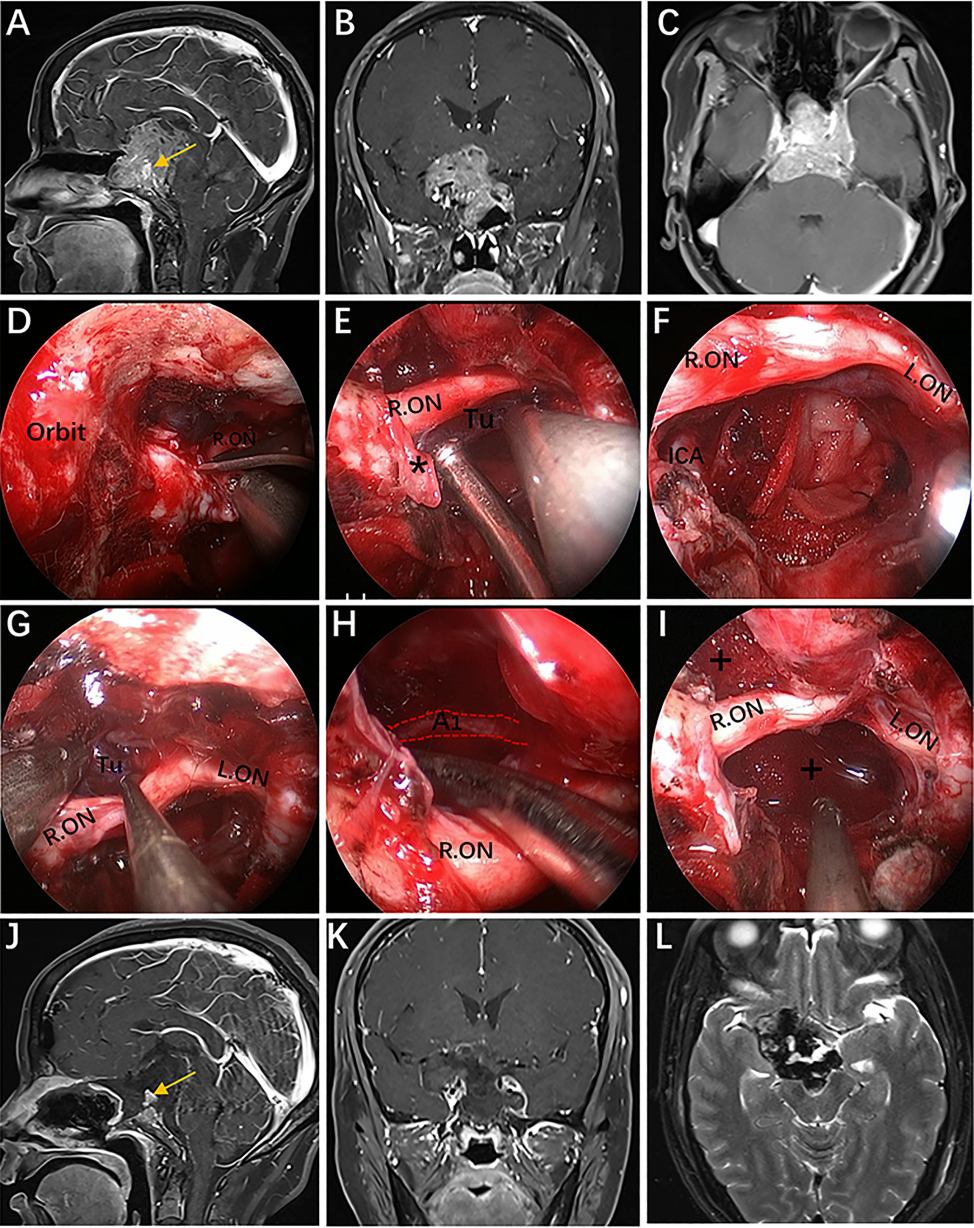
Case 6, a 45-year-old woman presented with visual loss and headache for 2 years. **(A–C)** Preoperative Gd-enhanced MRI showing a multilobulated pituitary adenoma with bilateral cavernous sinus invasion, inferior extension into the clivus, superior compressed the third ventricle, and significantly encasement of the ICA and its bifurcation. **(D–I)** Intraoperative images. **(D)** Opening of the optic nerve sheath. **(E, F)** Removal of the tumor below the right optic nerve using a bimanual microsurgical technique and endoscopic view after resection. **(G)** Expose the tumor above the optic nerve. **(H)** Removal of the tumor using angled suction instruments through the supraoptic corridor. The red dotted line indicates the course of the A1 segments of the anterior cerebral artery. **(I)** Endoscopic view after tumor resection. **(J–L)** Postoperative MRI showing total tumor removal. The yellow arrow represents the normal pituitary gland. * = optic nerve sheath; + = tumor cavity. The figure is available in color only online.

Our results validate the efficacy of the EESO and EEIO approaches in managing lesions involving the parasuprasellar area, as well demonstrated in our illustrations (**Figures 5**–**11**). In our experience, when tumors simultaneously invade the intrasellar, suprasellar and lateral to the parasuprasellar area, such as pituitary adenomas or craniopharyngiomas, the EEIO approach should be considered first. If the tumor is not safely exposed or still has an invisible portion, even after pulling it downward, the EESO approach should be selected to allow for additional exposure of the lateral tumor. When the lesion originates in the parasuprasellar area, such as ACP meningiomas or paraclinoid aneurysms, the EEIO approach can also be considered first to remove the lesion on the medial or lateral side of the paraclinoidal ICA. Similarly, if the lesion cannot be completely removed through the corridor below the ON, a combined EESO approach can be applied in most cases. It must be emphasized, however, that not all lesions involving this area are indications for EESO and EEIO approaches. Indeed, a primary TCA or staged operation may be indicated when the lesion involves the intracranial to the parasuprasellar area and is mainly located subdurally.

Of note, the use of the EESO or EEIO approach alone is rather rare and most often requires combination with the endoscopic endonasal midline approach and/or transcavernous approach. Different combinations of approaches can be selected according to the size and location of the lesion(s). For example, the transtuberculum/transplanum approach can be conveniently combined with the single EESO approach to provide better access to anterior cranial fossa meningiomas with lateral extension (case 1). Similarly, the combination of the transcavernous approach and EEIO approach has the potential for achieving complete resection of ACP meningiomas or pituitary adenomas involving the CS (cases 4, 6, 7 and 8). The EESO and EEIO approaches can be used as a complement to the midline approach and transcavernous approach and are extremely useful to access extensive pathologies for more complete resection while limiting morbidity.

Since the EESO and EEIO approaches require extensive removal of the skull base, strict cranial base reconstruction techniques for closure of the osteodural defect should be discussed. In our institution, we first use small pieces of autologous fat for intradural closure to eliminate the dead space. Then, we adopted the so-called sandwich technique, that is, two pieces of fascia lata or artificial dura were placed between the dura and bone as inlay substitutions and outside the bone as outlay substitutions, respectively. Finally, a vascularized pedicle nasoseptal flap harvested at the beginning of the procedure was positioned to cover the cranial defect, and each of the above layers was fixed with biological fibrin glue. However, it must be emphasized that, in the most lateral region of the cranial defect (where the ON stands), an inlay-overlay of dural substitutes will be difficult due to the irregularity of the osteodural defect. Therefore, autologous fat pieces should be placed across the intraextradural space.

### Graduated, Stepwise EEIO Approach

In our practice, the EEIO is a graduated, stepwise approach based largely on the lesion location, size and extent. Our anatomical study and clinical cases demonstrate how to assemble multiple surgical corridors to provide personalized access to complex parasuprasellar lesions. In Case 10, in which the left paraclinoid aneurysm was located just below the ON, successful clipping of the aneurysm was achieved using a pure EEIO approach (**Figure 6**).

Regarding Case 2, we found during the operation that a recurrent ACP meningioma was severely adhered to the ON and ICA and extended into the right OC. Thus, anterior clinoidectomy was applied, and the tumor, which was tightly attached to the ACP and the ICA bifurcation, was completely removed (**Figure 7**). It should be noted, however, that complete anterior clinoidectomy is not mandatory and that the extent of bony removal should be tailored to each case based on intraoperative need. If only the lateral region of the paraclinoidal ICA needs to be exposed or to obtain distal vascular control, partial anterior clinoidectomy should be considered; however, complete anterior clinoidectomy should be performed if the tumor involves the ACP and causes evident hyperplasia or the lesion has extended to involve the ICA bifurcation or even further. Such resection can reduce the risk of tumor recurrence. Most importantly, a corridor for accessing the lateral region of the supraclinoidal ICA is established while removing the involved ACP and the dura that envelops it. Nonetheless, this technique can only be implemented by experienced surgeons due to the complicated procedures and potential risks.

In Case 9, a lateral projecting paraclinoid aneurysm was encountered. In view of its position and orientation, we first performed anterior clinoidectomy to expose the lateral region of the paraclinoidal ICA. Then, the OA was dissociated and temporarily clipped, but the VEP changed, indicating that severing the OA would lead to serious visual impairment (**Figures 8E, H**). Finally, we attempted to expose the aneurysm neck between the OA and ON and successfully clipped the aneurysm through the ACP triangle (created by anterior clinoidectomy) (**Figures 8F, G**).

In Case 4, the tumor involved the intrasellar, suprasellar, CS, and encased the ICA and its bifurcation. The optic strut was drilled first, and the OA completely wrapped by the tumor was selectively dissected to further remove the hyperplastic ACP (**Figures 9F–H**). Thus, the tumor on the medial and lateral regions of the supraclinoidal ICA could be completely removed *via* an enlarged EEIO corridor (**Figures 9I, J**). This is the only case in our series, which preoperative DSA showed that the OA was not visible and the patient’s intraoperative VEP stabilized, the OA was sacrificed. As expected, the patient’s vision remained stable after surgery.

### Visual Outcomes

The special location of such lesions is often responsible for vision loss related to intracranial and/or intracanalicular ON involvement. Ten patients in our series presented with varying degrees of vision loss; thus, improvement and preservation of visual function is a priority for this surgery. Remarkably, in our series, visual improvement occurred in 7 patients but was unchanged in 4 patients, and only 1 patient with recurrent ACP meningioma developed visual deterioration. This demonstrates that gentle pulling of the ON during resection will hardly affect visual function under VEP monitoring. Postoperative visual deterioration has been mainly related to injury of the subchiasmatic perforators, providing the main blood supply to the optic chiasma (26). Accordingly, the potential risk of injuring visual acuity may not be increased by extra manipulation in the supraoptic region. Furthermore, while applying the EEIO approach, endoscopy provides early and direct visualization of the subchiasmatic perforators, allowing for adequate dissection and protection. Last but most importantly, this approach allows for direct 270° decompression of the intracanalicular ON (10) and prompt removal of the involved dura and hyperostotic bone. Nevertheless, as mentioned above, these procedures must be carried out in an extremely delicate and careful manner. When removing bone, the egg-shelling technique with continuous saline irrigation must be followed to prevent thermal injury to the ON. We believe that if sufficient decompression is performed without the risk of further injury, vision problems may be reversed.

### Limitations of the Study

The current study has several limitations that need to be considered. First, although no ICA or ON injury occurred in our series, these events are still our primary concern when managing parasuprasellar lesions. Second, cadaveric specimens are useful models to investigate surgical approaches, but they do not fully capture the clinical environment. Indeed, these corridors are relatively narrow in individuals who do not harbor such lesions. Finally, the learning curve is extremely steep and requires a high level of expertise in comprehensive skull base surgery, including both microsurgical cerebrovascular and endoscopic skills. Consequently, practice in the cadaver laboratory is mandatory to develop familiarity with these precise and meticulous operations before they are applied clinically.

## Conclusions

Based on our anatomical and surgical results, the EESO and EEIO approaches offer unique treatment options for well-selected lesions involving the parasuprasellar area. The approaches can be combined with the endoscopic endonasal midline approach and the transcavernous approach to remove extensive pathologies involving the intrasellar, suprasellar, sphenoid, and cavernous sinuses and even the bifurcation of the ICA. Our work for the first time pushes the boundary of the EEA lateral to the supraclinoidal ICA and ON.

## Data Availability Statement

The original contributions presented in the study are included in the article/supplementary material. Further inquiries can be directed to the corresponding author.

## Ethics Statement

The studies involving human participants were reviewed and approved by Institutional Ethics Committee of the First Affiliated Hospital of Nanchang University. The patients/participants provided their written informed consent to participate in this study.

## Author Contributions

TH contributed to the study conception and design. Material preparation and data collection were performed by YB, YY and LZ. Analysis of the data was performed by TH, YB, YY, LZ, SX, XW, HD, JW, LX, LY and BT. The first draft of the manuscript was written by YB. All authors commented on previous versions of the manuscript. All authors read and approved the final manuscript.

## Funding

This study was supported by the National Natural Science Foundation of China (grant nos. 82060246 and 81460381), the Ganpo555 Engineering Excellence of Jiangxi Science and Technology Department (2013), and the Key Research and Invention Plan of Jiangxi Science and Technology Department (20192BBG70026).

## Conflict of Interest

The authors declare that the research was conducted in the absence of any commercial or financial relationships that could be construed as a potential conflict of interest.

## Publisher’s Note

All claims expressed in this article are solely those of the authors and do not necessarily represent those of their affiliated organizations, or those of the publisher, the editors and the reviewers. Any product that may be evaluated in this article, or claim that may be made by its manufacturer, is not guaranteed or endorsed by the publisher.
